# Artificial Intelligence and Amikacin Exposures Predictive of Outcomes in Multidrug-Resistant Tuberculosis Patients

**DOI:** 10.1128/AAC.00962-16

**Published:** 2016-09-23

**Authors:** Chawangwa Modongo, Jotam G. Pasipanodya, Beki T. Magazi, Shashikant Srivastava, Nicola M. Zetola, Scott M. Williams, Giorgio Sirugo, Tawanda Gumbo

**Affiliations:** a Division of Infectious Diseases, University of Pennsylvania, Philadelphia, Pennsylvania, USA; b Botswana-University of Pennsylvania Partnership, Gaborone, Botswana; c Center for Infectious Diseases Research & Experimental Therapeutics, Baylor Research Institute, Baylor University Medical Center, Dallas, Texas, USA; d Department of Medical Microbiology, University of Pretoria, Pretoria, South Africa; e Department of Medicine, University of Botswana, Gaborone, Botswana; f Department of Genetics, Geisel School of Medicine, Dartmouth College, Hanover, New Hampshire, USA; g Centro di Ricerca, Ospedale San Pietro Fatebenefratelli, Rome, Italy; h Department of Medicine, University of Cape Town, Observatory, Cape Town, South Africa

## Abstract

Aminoglycosides such as amikacin continue to be part of the backbone of treatment of multidrug-resistant tuberculosis (MDR-TB). We measured amikacin concentrations in 28 MDR-TB patients in Botswana receiving amikacin therapy together with oral levofloxacin, ethionamide, cycloserine, and pyrazinamide and calculated areas under the concentration-time curves from 0 to 24 h (AUC_0–24_). The patients were followed monthly for sputum culture conversion based on liquid cultures. The median duration of amikacin therapy was 184 (range, 28 to 866) days, at a median dose of 17.30 (range 11.11 to 19.23) mg/kg. Only 11 (39%) patients had sputum culture conversion during treatment; the rest failed. We utilized classification and regression tree analyses (CART) to examine all potential predictors of failure, including clinical and demographic features, comorbidities, and amikacin peak concentrations (*C*_max_), AUC_0–24_, and trough concentrations. The primary node for failure had two competing variables, *C*_max_ of <67 mg/liter and AUC_0–24_ of <568.30 mg · h/L; weight of >41 kg was a secondary node with a score of 35% relative to the primary node. The area under the receiver operating characteristic curve for the CART model was an R^2^ = 0.90 on posttest. In patients weighing >41 kg, sputum conversion was 3/3 (100%) in those with an amikacin *C*_max_ of ≥67 mg/liter versus 3/15 (20%) in those with a *C*_max_ of <67 mg/liter (relative risk [RR] = 5.00; 95% confidence interval [CI], 1.82 to 13.76). In all patients who had both amikacin *C*_max_ and AUC_0–24_ below the threshold, 7/7 (100%) failed, compared to 7/15 (47%) of those who had these parameters above threshold (RR = 2.14; 95% CI, 1.25 to 43.68). These amikacin dose-schedule patterns and exposures are virtually the same as those identified in the hollow-fiber system model.

## INTRODUCTION

Multidrug-resistant tuberculosis (MDR-TB) requires therapy with second-line antituberculosis drugs such as aminoglycosides (1). Together with 8-methoxyquinolones such as moxifloxacin, aminoglycosides form the so-called optimized background regimen backbone upon which newer agents are added for the treatment of MDR-TB (1–3). Amikacin is among the most commonly used aminoglycosides. However, the amikacin exposures that could delineate patients who will respond to therapy and those who will not respond to therapy are unknown. This is important to know since we have identified amikacin concentrations associated with toxicity: once those associated with optimal outcomes are known, clinicians and TB programs will have a therapeutic window within which to dose (4). In the meantime, we have also examined amikacin monotherapy in the hollow-fiber system model of TB (HFS-TB), described in the accompanying article, and identified a peak concentration (*C*_max_)-to-MIC ratio of 10.13 as being most closely linked to efficacy (*r*^2^ > 0.99), which would translate to a *C*_max_/MIC ratio of 75 in the serum (5). This was followed closely by a area under the concentration-time curve from 0 to 24 h (AUC_0–24_)-to-MIC ratio of 102.74 (*r*^2^ = 0.98). While the HFS-TB has demonstrated robust accuracy for clinical events (6–8), a clinical study was nevertheless still needed to confirm the findings and to identify the optimal amikacin exposures in patients.

A common concern in translating results from the laboratory to the clinic is that antibiotic exposures associated with optimal effects in preclinical models of monotherapy may not be the same as those for the same drug in patients who are treated with combination therapy. Indeed, one commonly expressed opinion is that since TB is an intracellular disease (which is not even true about cavitary pneumonia), standard pharmacokinetics/pharmacodynamics (PK/PD) do not apply. In addition, in HFS-TB monotherapy studies, optimal exposure is defined as the exposure mediating 90% of maximal kill (EC_90_) or sometimes EC_80_, while in patients it would be the exposure below which high proportions of patients fail therapy, often in combination therapy regimens. It is unclear if the two exposures would be the same. In order to investigate this crucial question, we measured amikacin concentrations in MDR-TB patients in Botswana and utilized these concentrations to identify amikacin pharmacokinetic parameters for each patient. We sought to determine if amikacin concentrations play a role in microbial outcomes at all and, if so, what the exposures associated with optimal efficacy were. We utilized agnostic machine learning algorithms to identify significant predictors of outcome by searching through all possible clinical predictors *in toto*, including various amikacin exposure measures. These artificial intelligence (AI) methods search for the “needle” in the proverbial “haystack” of clinical factors, determine if each is even relevant, and, if relevant, automatically calculate the drug exposure threshold predicting the outcome (9–12). These AI methods are nonparametric, are distribution free, and do not depend on the investigator prespecifying the importance of a potential predictor such as drug exposure and thus pressing a thumb on the scale. However, these methods are not for hypothesis testing, as is the classic role of frequentist statistics, but rather are for hypothesis generation. Two of these AI methods, classification and regression tree analysis (CART) and multivariate adaptive regression splines (MARS), have been powerful tools for identifying the nonlinear and higher-order interactions inherent in relationships between treatment variables and TB outcome and have outperformed standard frequentist statistical inferences (10, 11, 13–15). Here, we used CART to identify and rank in order of importance the predictors of good outcome in MDR-TB patients in Botswana. The CART findings were then used in hypothesis testing to determine if patients identified by the predictors were more likely to fail therapy.

## MATERIALS AND METHODS

We recently recruited 28 MDR-TB patients in Botswana who were treated with a standardized amikacin-containing regimen. The patients were recruited for the purpose of identifying amikacin concentrations predictive of ototoxicity, based on high rates of ototoxicity in Botswana. Thus, the sample size was adequate for that study. The regulatory and ethical details, recruitment schedule, audiometry results, and the ototoxicity results have been published separately (4, 16). The clinical study was approved by the Human Research Development Committee at the Ministry of Health, Botswana, and the University of Pennsylvania Institutional Review Board. Based on the protocol for treatment of MDR-TB patients by the Ministry of Health in Botswana, all patients received oral levofloxacin, ethionamide, cycloserine, and pyrazinamide. Amikacin was administered 5 days per week via the intramuscular route at doses of 15 to 25 mg per kg (maximum dose of 1,000 mg per day); injections were discontinued 4 months after culture conversion.

Blood was drawn from each patient, and amikacin concentrations were measured on Cobas Integra systems, as described before (4). Compartmental pharmacokinetic analyses were then performed for each patient, and the pharmacokinetics of amikacin were best described by a two-compartment model. With these parameters, an AUC_0–24_ was calculated for each patient. In the end, three concentration measures were available for each patient, i.e., an observed *C*_max_ and an observed trough as well as the calculated AUC_0–24_. Patients were followed closely by the team for audiometry. These patients were also followed by the same team for microbial outcomes based on the standard clinical monitoring for MDR-TB prescribed by the Ministry of Health in Botswana. Patients were followed monthly for microbial outcomes using the mycobacterial growth indicator tube (MGIT). For the purposes of this study, we defined sputum conversion as one negative sputum culture on follow-up in these MDR-TB patients. Patients were followed for several months until sputum conversion or cessation of therapy.

Classification and regression tree analysis (CART) was used to identify predictors associated with sputum culture conversion during amikacin therapy, the nonlinear interaction between the predictors, and their importance ranking. The main outcome examined was sputum conversion. All the clinical features, including patient demographic and clinical factors, comorbid conditions such as HIV infection, and treatment, as well as amikacin concentrations, including *C*_max_, trough concentrations, AUC_0–24_, and dose in milligrams and in milligrams per kilogram, were included as potential predictors. CART assigns a measure of predictive importance to each potential predictor, entailing both marginal and interaction effects involving this variable. Variable importance is a unique tool found with machine-learning approaches that can be used for ranking and selecting the most influential predictors in the final model. Because CART uses recursive partitioning of data into homogenous groups, it is resistant to collinearity, which is especially helpful for situations in which the number of predictors is relatively large compared to the number of observations (as was the case with this clinical study). The detailed steps followed in CART, including the pruning of trees, and final selection of the model were as described by us in detail in the past (4, 11, 17–19). We performed a 10-fold cross validation, an exercise in which the whole data set is randomly split into different smaller test data sets and CART performed, in order to determine model performance and predictive accuracy in future independent samples.

Work in the hollow-fiber model of tuberculosis and in MDR-TB patients treated with amikacin, as well as measures of drug concentration in combination therapy by Mpagama et al., has demonstrated that MIC affects patient response (5, 10, 20). Indeed, this is a standard tenet of PK/PD theory (21, 22). We did not have MICs for isolates in our Botswana patients. However, in order to get an idea of the MIC distribution in the same geographic locale, we identified amikacin MICs in 62 MDR-TB isolates from Gauteng Province, which is only 170 km from Gaborone. The MICs were from clinical samples from MDR-TB patients and were identified in Gauteng Province using Sensititre plate assays (23).

Finally, since CART is used not for hypothesis testing (24) but for hypothesis generation, we subjected the CART findings to standard hypothesis testing. We examined outcomes in patients grouped by predictors identified by CART for measures of association such as relative risk (RR) and 95% confidence intervals (CI). Comparison between patient group medians were made using the Kruskal-Wallis rank test, while Fischer's exact test was used to compare proportions. For MIC distribution, we tested for normality using the D'Agostino-Pearson normality test.

## RESULTS

**FIG 1:**
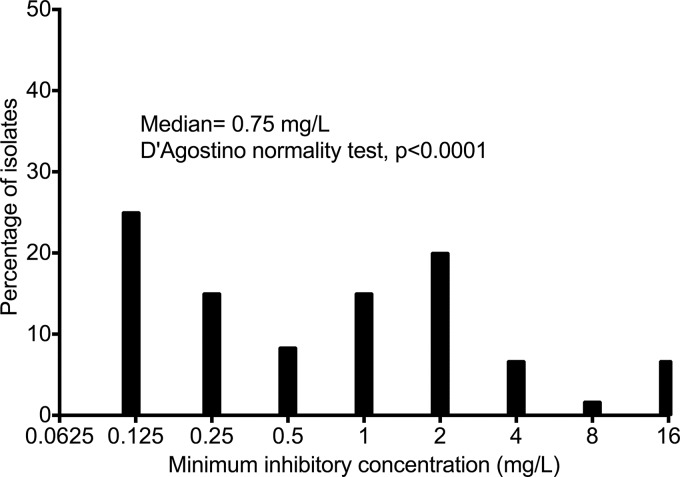
Amikacin MIC distribution in 62 MDR-TB isolates. The amikacin MICs from Gauteng Province, adjacent to Gaborone, Botswana, are shown. The MICs were not normally distributed; however, those for ∼50% of all isolates were within one to two dilutions of 1 mg/liter.

The amikacin MIC distribution from MDR-TB patients is shown in Fig. 1. The figure shows that this was not a normal distribution and had a D'Agostino normality test *P* value of <0.0001. The median MIC was 0.75 mg/liter, the MIC_50_ was 1.0 mg/liter, and the MIC_90_ was 2.0 mg/liter. The MIC range was from 0.125 mg/liter to >16 mg/liter.

The clinical characteristics of the MDR-TB patients in Botswana, including doses of amikacin used to treat the patients, are shown in Table 1. The table shows that the median duration of therapy was 184 (range, 28 to 866) days, and the median dose was 17.30 (range, 11.11 to 19.23) mg/kg/day. The compartmental pharmacokinetic parameter estimates in the patients have been published previously. The median peak amikacin concentrations, AUCs, and trough concentrations are also shown in Table 1. The relationship between peak and AUC was linear (Pearson's *r* = 0.52), as were those for peak versus trough concentration (*r* = 0.28) and AUC versus trough concentration (*r* = 0.40). Thus, the correlation was low.

**TABLE 1:** Comparison of clinical factors in patients with and without culture conversion

Parameter	Value^*a*^ for patients with:		*P* value
	No culture conversion (*n* = 17)	Culture conversion (*n* = 11)	
Age, yr	42 (17–81)	42 (16–69)	0.693
Gender			
Female	7 (41)	5 (45)	0.823
Male	10 (59)	6 (55)	
HIV test			
Negative	10 (59)	6 (55)	0.823
Positive	7 (41)	5 (45)	
Previous TB treatment			
Yes	5 (29)	5 (45)	0.387
No	12 (71)	6 (55)	
Wt, kg^*b*^	51.35 (26–64)	52 (39–70)	0.786
Amikacin therapy duration, days	172.5 (28–320)	207.5 (65–866)	0.041
Amikacin dose, mg/kg	17.44 (13.70–19.23)	17.01 (11.11–19.23)	0.961
Amikacin *C*_max_, mg/liter	49.42 (22.02–65.59)	49.42 (25.94–76.95)	0.493
Amikacin AUC_0–24_, mg · h/liter	556.91 (241.50–988.71)	599.56 (446.34–766.91)	0.371
Amikacin trough concn, mg/liter	0.64 (0–11.89)	0 (0–4.92)	0.338

a Values are either number (%) of patients or median (range).

b Data for some patients are missing.

Of the 28 patients, 11 (39%) had sputum culture conversion during treatment. First, a comparison between patient sputum conversion group medians was made using the Kruskal-Wallis rank test, while the likelihood ratio test was used to compare proportions. The results are shown in Table 1. Only duration of therapy was found to be significantly associated with outcome, with longer duration of therapy associated with better sputum conversion on the univariate analysis. Amikacin *C*_max_, AUC, and trough median concentration were not significantly different between patients who had sputum conversion and those who did not.

CART identified amikacin *C*_max_, AUC, and weight as driving sputum conversion. The primary node was made of two competing variables, *C*_max_ and AUC_0–24_, which had the variable importance score of 100%; weight was a secondary node with a score of 35% relative to the primary node. The threshold *C*_max_, AUC_0–24_, and weight were 67 mg/liter, ≥568.30 mg · h/liter, and >41 kg, respectively. The trough concentration ranked low and was not an important predictor of outcome. There was an interaction between amikacin AUC_0–24_ and weight, which means that weight modified the effect of AUC_0–24_ on sputum conversion. The area under the receiver operating characteristic curve (AUROC) for the model was an R^2^ = 0.90 on posttest.

When these CART thresholds were used to compare outcomes in the patients weighing >41 kg, based on use of the CART trees as decision trees, sputum conversion in patients with an amikacin *C*_max_ of ≥67 mg/liter was 3/3 (100%), versus 3/15 (20%) in those with a *C*_max_ of <67 mg/liter, a relative risk of 5.00 (95% CI, 1.82 to 13.76; *P* = 0.025). Sputum conversion in patients weighing >41 kg who had an amikacin AUC of ≥568.30 mg · h/liter was 7/14 (50%), versus 1/8 (12.5%) in those with an AUC of <568.30 mg · h/liter, a relative risk of 4.50 (95% CI, 0.66 to 30.73; *P* = 0.086). In patients who had both the amikacin *C*_max_ and AUC below the threshold, 7/7 (100%) patients did not have sputum conversion, compared to 7/15 (47%) of patients who had these parameters above the threshold, a relative risk of 2.14 (95% CI, 1.25 to 43.68; *P* = 0.023) for therapy failure. One patient whose weight variable was missing did not achieve sputum conversion. Only 2/5 patients who weighed <41 kg had both a low amikacin *C*_max_ and a low AUC_0–24_; they too failed therapy.

## DISCUSSION

First, in the HFS-TB, we found concentration-dependent efficacy with the *C*_max_/MIC ratio driving amikacin monotherapy efficacy, closely followed by the AUC_0–24_/MIC ratio; trough-based exposures were vanishingly unimportant compared to *C*_max_/MIC ratio, with a relative likelihood for the trough/MIC ratio that was <1/1,000 of that for the *C*_max_/MIC ratio (5). Here, we show that in MDR-TB patients, *C*_max_ and AUC_0–24_ were the parameters that drove efficacy of amikacin combination therapy, with *C*_max_ having a significantly increased risk ratio of failure when it was low. Trough concentration was unimportant and not ranked. Thus, the patterns seem to be the same in the HFS-TB monotherapy studies as for MDR-TB patients on combination therapy. Given that experimental design is optimized in the HFS-TB, with colinearity of exposures broken based on the design, the indices were identified using linear analyses and Akaike information criteria, while in the clinic we used AI algorithms that utilize nonlinear analyses. Linear analyses are characterized by the principle of superimposition: a problem can be broken into its parts, and the solutions can be added and add to the whole. Thus, even “nonlinear” regression models, such as the inhibitory sigmoid *E*_max_ model, are by this definition linear analyses. On the other hand, in nonlinear science, there are recognized interactions of predictors *in toto* that change when each predictor is separated from the whole and considered alone. Breaking problems into smaller portions, finding solutions, and then adding them up does not add up to the observed net response, i.e., does not follow the superposition principle. Thus, in nonlinear analyses, the behavior of components when they are part of the whole (i.e., with all other factors) is best be characterized by testing all parameters *in toto*. Given that physiological systems are nonlinear systems, clinical data are best analyzed using nonlinear science approaches. As an example, we found that weight interacted with AUC_0–24_ but that each also affected sputum conversion directly, while the effects of *C*_max_ above and below the specific weight of 41 kg differed. In summary, parametric regression models that utilized linear analyses in the HFS-TB identified patterns similar to those in patients who were analyzed based on nonlinear analyses.

Second, it is self-evident from our results that pharmacokinetic variability plays an important role in failure of amikacin therapy in the optimized background regimen for MDR-TB. One recent advance in the treatment of drug-susceptible TB, first identified in the HFS-TB and computer-aided clinical trial simulations, has been the effect of pharmacokinetic variability as a major cause of therapy failure and acquired drug resistance (11, 25–28). This concept might modify the focus from tuberculosis program factors such as directly observed therapy to development of clinical decision-making tools for monitoring drug concentrations and redosing to optimize outcomes (29). The mechanism by which this variability drives failure is simply poor microbial kill by subtherapeutic drug concentrations in patients on recommended doses. Since pharmacokinetic variability is encountered for virtually every antibiotic we have examined, we propose that the same process will apply to all other second-line anti-TB compounds.

Third, we now have identified the amikacin threshold concentrations that predict optimal microbial outcomes in MDR-TB patients. In the HFS-TB, we identified the *C*_max_/MIC ratio of 10.13 as the EC_90_, based on inhibitory sigmoid *E*_max_ model regression (5). On the other hand, CART is a more agnostic algorithm and essentially found the *C*_max_ for which there is homogeneity for sputum conversion versus non-sputum conversion. The two definitions thus describe two different types of response. Using CART, we identified a *C*_max_ of 67 mg/liter in serum as the threshold. Given the MIC distribution shown in Fig. 1, described by a median of 0.75 mg/liter, and that half the isolates were within one tube dilution MIC of 1.0 mg/liter (which is also the MIC_50_), the serum *C*_max_/MIC ratio is expected to be 67 to 89. Furthermore, given the amikacin serum-to bronchial secretion ratio of 0.135 as well as negligible protein binding, this calculates to a pulmonary *C*_max_/MIC ratio of 9.05 to 12.02 (30). This value is in the same range as the HFS-TB-derived EC_90_ *C*_max_/MIC ratio of 10.13 (30). Similarly, if one used the CART-identified AUC of ≥568.30 mg · h/liter in the serum, this translates to an AUC_0–24_/MIC ratio of 76.72 to 102.29 in the lung, which is reasonably close to the HFS-TB derived AUC_0–24_/MIC ratio of 102.74 (95% CI, 77.7 to 127.8) (5). Thus, amikacin exposure thresholds associated with a good response in combination therapy in MDR-TB are within the same range as the EC_90_ derived in HFS-TB experiments. These amikacin exposures are the targets for dosing that tuberculosis programs should aim to achieve for maximal efficacy. These will need to be counterbalanced with a dosing strategy that minimizes toxicity (4).

There are some limitations in our study. Different combination drugs are given for various durations during treatment of MDR-TB, which could affect generalizability to other settings. In our case, all patients were treated with same standard combination regimen. Thus, larger prospective studies will be required to examine the amikacin thresholds identified in this study in the context of specific multidrug MDR-TB treatment regimens and by taking into account drug concentrations of companion drugs. Closely related is the lack of data on amikacin MICs for each patient matched to the response; similarly, MICs of companion drugs would have been informative. These concentrations, however, were not measured, which could limit the generalizability of our findings. To partially mitigate this, we examined the most common MICs that would be encountered in these patients, as shown in Fig. 1. Moreover, in the hollow-fiber system, we took into account the Mycobacterium tuberculosis MIC and still identified an optimal exposure within the same range as in these patients. This suggests that our results could have meaning beyond the specific data set. A common critique of these types of studies is about sample size. However, we reiterate that the AI methods are there for hypothesis generation. Even with that limitation, nevertheless, when the CART-derived cutoff points were examined in hypothesis testing for the relative risk of failure, the sample size was adequate to detect statistically significant differences. Second, there is often concern about collinearity between the drug concentrations, which would make a deliberate dose fractionation design a better approach. However, in our data set, correlations were weak, with the highest *r* being only 0.52. Moreover, we used experimental design to break collinearity in the HFS-TB and identified similar dose schedules as best linked to efficacy as in the patients (5). Furthermore, CART was designed in part, to break collinearity, which it evidently succeeded in doing. Third, the definition of sputum conversion we used was one negative sputum liquid culture. Others have used two consecutive cultures; however, one negative culture has also been used.

In summary, we found that amikacin peak concentration and AUC, as well as patient weight, were most predictive of sputum conversion in MDR-TB patients. These are similar to patterns in the HFS-TB described in the accompanying paper (5). The threshold concentrations should be considered dosing targets for improving sputum conversion in MDR-TB patients on amikacin-based regimens for TB programs.
